# Designing a Conserved Immunogenic Peptide Construct from the Nucleocapsid Protein of *Puumala orthohantavirus*

**DOI:** 10.3390/v16071030

**Published:** 2024-06-26

**Authors:** Ayushi Sehgal, Diksha Sharma, Neha Kaushal, Yogita Gupta, Ekaterina Martynova, Emmanuel Kabwe, Sara Chandy, Albert Rizvanov, Svetlana Khaiboullina, Manoj Baranwal

**Affiliations:** 1 Department of Biotechnology, Thapar Institute of Engineering and Technology, Patiala 147001, India; ayushisehgal0703@gmail.com (A.S.); sdiksha946@gmail.com (D.S.); kneha0563.nk@gmail.com (N.K.); yogita.gt@gmail.com (Y.G.); 2 Institute of Fundamental Medicine and Biology, Kazan (Volga Region) Federal University, Kazan 420008, Russia; martynova85@gmail.com (E.M.); emmanuelkabwe@ymail.com (E.K.); sv.khaiboullina@gmail.com (S.K.); 3 Childs Trust Medical Research Foundation (CTMRF) Kanchi, Chennai 600034, India; dr.sarachandy@ctmrf.org

**Keywords:** orthohantavirus, hemorrhagic fever with renal syndrome (HFRS), Nucleocapsid protein, Multiepitope, HLA-peptide interactions

## Abstract

*Puumala orthohantavirus* (PUUV) is an emerging zoonotic virus endemic to Europe and Russia that causes nephropathia epidemica, a mild form of hemorrhagic fever with renal syndrome (HFRS). There are limited options for treatment and diagnosis of orthohantavirus infection, making the search for potential immunogenic candidates crucial. In the present work, various bioinformatics tools were employed to design conserved immunogenic peptides containing multiple epitopes of PUUV nucleocapsid protein. Eleven conserved peptides (90% conservancy) of the PUUV nucleocapsid protein were identified. Three conserved peptides containing multiple T and B cell epitopes were selected using a consensus epitope prediction algorithm. Molecular docking using the HPEP dock server demonstrated strong binding interactions between the epitopes and HLA molecules (ten alleles for each class I and II HLA). Moreover, an analysis of population coverage using the IEDB database revealed that the identified peptides have over 90% average population coverage across six continents. Molecular docking and simulation analysis reveal a stable interaction with peptide constructs of chosen immunogenic peptides and Toll-like receptor-4. These computational analyses demonstrate selected peptides’ immunogenic potential, which needs to be validated in different experimental systems.

## 1. Introduction

*Puumala orthohantavirus* (PUUV) belongs to the genus orthohantavirus and the family *Hantaviridae* [1]. PUUV circulates in small rodents, and it can cause outbreaks in endemic regions [2]. In rodents, infection is lifelong-lasting and asymptomatic [3]. In contrast, human infection could lead to various levels of severity in the form of diseases like hemorrhagic fever with renal syndrome (HFRS) [3,4]. Symptoms of HFRS include thrombocytopenia, high fever, headache, and acute kidney injury (AKI) [5]. Severe AKI is the primary cause of death in HFRS patients [6]. Several species within the genus orthohantavirus are known to cause diseases in humans, which are the *Hantaan virus* (HNTV), *Seoul virus* (SEOV), *Puumala virus* (PUUV), and *Dobrava-Belgrade virus* (DOBV) [7]. PUUV infection is endemic to Europe and the western part of Russia, where the number of cases could be as high as 10,000 annually [8,9]. Infection is transmitted through inhaling aerosolized rodent excreta or consuming food contaminated with PUUV [10].

PUUV is a single-stranded, negative-sense enveloped RNA virus with a genome that comprises small (S), medium (M), and large (L) segments [1]. These segments encode the nucleocapsid (N) protein, the envelope glycoprotein (G1 and G2), and the RNA-dependent RNA polymerase (RdRp) protein, respectively [11]. The N protein is multifunctional and contributes to the intracellular transport and assembly of the viral genome. Also, it binds to host cell proteins and promotes replication [12]. Numerous studies have identified the N protein as highly conserved among orthohantaviruses, making it a valuable target for diagnosing and developing vaccine candidates [11]. Studies support this assumption; the reactivity of recombinant PUUV N proteins was demonstrated in HFRS convalescent samples [13]. It was reported that anti-N protein IgM was detected in the serum at the acute phase of infection, and IgG was detected in convalescent sera at greater levels. In contrast, IgA was present at higher levels in both acute and convalescent phases [13]. Antibody reactivity with N protein was also long-lasting and could be used to diagnose previous exposure to PUUV. The N protein exhibits strong cross-protection across many geographical regions, and it is a crucial component in developing comprehensive vaccines targeting hantavirus infection [14].

In recent years, significant progress has been made in identifying potentially immunogenic peptides using the immunoinformatic approach, which has shown promising results in various experimental systems [15]. Various immunoinformatic methods were employed to identify immunogenic peptides containing multiple epitopes of SARS-CoV-2 [16], DENV [17], Ebola virus [18], H1N1 influenza virus [19,20], CanineCV [21], Zika virus [22], and hantavirus [23]. Computational identification of immunogenic peptides of Zika virus, SARS-CoV-2, Hantaan virus, and influenza virus has yielded favorable results in several experimental investigations [24,25,26]. These findings illustrate the effectiveness of combining immunoinformatic methods with experimental validation to identify potential immunogenic candidates for vaccine development or diagnosis of different viruses. In this study, we have applied various immunoinformatic tools to select three conserved immunogenic peptides containing multiple T and B cell epitopes of PUUV N protein. Population coverage and molecular docking analyses suggest that the chosen peptides can interact with diverse HLA alleles, suggesting their potential to be presented in HLA alleles, leading to the activation of T cells. The peptide constructs generated by linking immunogenic peptides and linkers have shown stable interactions with Toll-like receptor-4 (TLR-4) during molecular docking and simulation assessment. Interestingly, the generated immunogenic peptide construct consists of multiple T cell (CD8^+^ and CD4^+^) epitopes, and B cell epitopes interact with diverse HLA alleles and TLR-4.

## 2. Materials and Methods

### 2.1. Sequence Retrieval and Conserved Peptide Identification

The PUUV N protein sequences, consisting of 433 amino acids, were obtained from the NCBI database in FASTA format. Jalview software version 2.11.3.3 [27] was used with a threshold value of 100 to eliminate redundancy. The selected N protein sequences were aligned using the Multiple Sequence Comparison by Log-Expectation (MUSCLE) tool within the MEGA X (Molecular Evolutionary Genetics Analysis) software version 11.0.13 [28]. The Antigen Variability Analyzer (AVANA) tool [29], which utilizes the Ethereum algorithm, was employed to identify the conserved regions within the N protein. This analysis aimed to identify regions with minimum variability across different PUUV strains.

### 2.2. Prediction of T and B Cell Epitopes

The NetMHCpan-4.1 server [30] was used for CD8^+^ T cell epitopes (HLA class I) with a threshold value of 0.5 and 2 for strong and weak binders, respectively. NetMHCIIpan-4.0 [31] was employed to predict CD4^+^ T cell epitopes (HLA class II), keeping a threshold value of 1 and 5 (% rank) for strong and weak binders, respectively. Class II MHC has an open binding groove; hence, the length of CD4^+^ epitopes was set to fifteen. In addition to T cell epitope prediction, B cell epitopes were predicted using the ABCpred epitope prediction tool [32]. This tool utilizes a specific algorithm that uses artificial neural networks to identify potential B cell epitopes within the protein sequence.

### 2.3. Screening of Peptide for Antigenicity and Allergenicity

The selected peptides were screened to evaluate for their antigenicity and allergenicity properties. Vaxijen v2.0 server [33] was utilized to assess the antigenicity, which employs auto cross-covariance (ACC) for virus antigens, keeping a threshold of 0.4 to define potential antigens. The AlgPred tool [34] utilizes the Motif Alignment and Search Tool for predicting IgE epitopes.

### 2.4. Population Coverage Analysis

The IEDB population tool was utilized [35] to assess the expected potential of peptides to respond across various geographical regions. This population coverage analysis tool included the alleles of sixteen distinct geographical regions, which included 115 countries and 21 different ethnicities. It covers six out of seven continents except Antarctica. A list of HLA alleles that displayed an affinity for the identified peptides was compiled and used as input for this population analysis tool.

### 2.5. Molecular Docking

Molecular docking was employed to investigate the interaction between human leukocyte antigen (HLA) molecules and the identified peptides/epitopes. For this purpose, the HPEP dock server [36] was utilized. High-resolution crystal structures of ten each of class-I and class-II HLA belonging to the most common HLA allele supertype, bound with their respective naturally bound peptides (NP), were obtained from the Protein Data Bank (PDB). Using Discovery Studio software 2021, the naturally bound peptides were extracted from the HLA crystal structures. Refinement and energy minimization of both the naturally bound peptides and the HLA structures were performed using tools, such as the Galaxy server [37], 3D refine [38], and Chimera [39]. To establish a reference for docking, the naturally bound peptide of each HLA molecule was separated and redocked to obtain a reference binding energy. The 3D structures of the identified peptides/epitopes were generated using the Modpep tool [40]. These peptides were then subjected to refinement and energy minimization using the 3D refine and Chimera tools. The resulting docked complex was visualized using the Pymol tool to analyze the peptide–HLA interactions.

The 3D structure of Toll-like receptor-4 (TLR-4) (PDB ID: 4G8A) was downloaded from the PDB database. TLR-4 was docked with an immunogenic peptide construct using the Cluspro 2.0 server [41], a protein–protein docking server based on rigid docking. The docking score was compared to that of a naturally bound peptide. The naturally bound peptide used here was Lymphocyte antigen 96, which was bound along with the structure of TLR-4 that was downloaded from PDB (PDB ID-4G8A). The naturally bound peptide was first separated and then redocked with TLR-4, which was used as the control to compare with peptide construct docking with TLR-4. PYMOL was used to obtain a 3D docking pose and locate the hydrogen bond interactions, whereas a 2D plot exhibiting various residues involved in hydrogen bonds was obtained by using LIGPLOT^+^.

### 2.6. Design of an Immunogenic Peptide Construct and Prediction of Discontinuous B Cell Epitopes in Construct

The Robetta server was used to model an immunogenic peptide construct’s three-dimensional structure containing selected peptides and the flexible linkers ‘GGGGS’. The Robetta server provides automated structure prediction and analysis tools to estimate the protein structure from genetic data [42]. The Galaxy refine tool refined the model, using an ab initio method and terminus modelling for the refinement [42]. The verified model was evaluated using Verify 3D, ERRAT, and PROCHECK, which included the analysis of the Ramachandran plot [43]. The verified 3D plot assesses the compatibility of the model protein structure with its amino acid sequence. ERRAT evaluates the quality of the 3D structure, aiming for a model with an overall quality factor of ≥93.97%. The Psipred [44] tool was used to predict the secondary structure of the generated construct. Furthermore, the discontinuous B cell epitopes in the construct were predicted using the Discotope 2.0 tool [45]. These epitopes were then mapped in the three-dimensional structure of the immunogenic construct.

### 2.7. Molecular Dynamic Simulation

GROMACS v2021.3 [46] was used to perform molecular dynamics (MD) simulations on TLR-4, a peptide construct, and their docked complex. The molecular structure and arrangement of each molecule and their complexes were created using the charmm27 force field within the TIP3P water box. The molecules in question were placed within the triclinic box using the TIP3P water model. The introduction of Na+ and Cl- ions neutralized the overall charge of the system. This was followed by a process of energy minimization spanning fifty thousand steps. Afterwards, the system proceeded through equilibration in two stages: first in an ensemble with a constant number of particles, volume, and temperature (NVT), and then in an ensemble with a constant number of particles, pressure, and temperature (NPT) for a duration of one hundred picoseconds. A molecular dynamics (MD) simulation was conducted for a duration of 100 nanoseconds to investigate the root mean square fluctuation (RMSF), root mean square deviation (RMSD), and radius of gyration (Rg).

## 3. Results

### 3.1. Identification of Conserved Peptide Fragments

One hundred twenty-three complete N protein sequences of PUUV isolated from HFRS were downloaded from the NCBI database in FASTA format. The sequences available by 9 April 2024 were included in this analysis. After removing redundant sequences, a total of sixty-four unique sequences were selected to identify the conserved peptides. Initially, ten peptides with 90% conservancy were identified using AVANA. Next, these peptides were BLAST-analyzed to determine their identity (seven in nine consecutive amino acids) with human proteins. Parts of five conserved peptides have shown identity with human proteins, such as human immunoglobulin heavy chain, APE2 protein, Zinc finger proteins, and Myosin (MYH6) proteins (Supplementary Table S1). Thirteen conserved peptides were obtained after excluding the part of peptides resembling human proteins (Table 1).

### 3.2. Peptides Containing T and B Cell Epitopes

The thirteen conserved N peptides were used as input to the NetMHCpan 4.1 server for predicting CD8^+^ T cell epitopes, considering 2915 class I HLA alleles. A total of 74 strong binder CD8^+^ T cell epitopes were predicted (Supplementary Table S2). Seven peptides containing more than one overlapping CD8^+^ T cell epitope were selected (Table 2).

The seven selected peptides were then used as input for CD4^+^ T cell epitope prediction for 681 alleles for class II using the NetMHCIIpan 4.0 server. In total, 26 CD4^+^ T cell epitopes with strong binding with HLA alleles were obtained (Supplementary Table S3), and six peptides containing overlapping CD4^+^ epitopes were selected (Table 3). Next, the presence of B cell epitopes was analyzed in these six N peptides using the ABCpred tool. Among them, three peptides (P1, P2, and P3) contained B cell epitopes. These peptides, containing multiple T and B cell epitopes, were selected for further investigation (Table 4). Furthermore, the antigenic score (Vaxijen v2.0) of these three peptides was more than 0.4, confirming the antigenic nature of the peptides. These peptides were also predicted to be non-allergens.

### 3.3. Peptides Binding to HLA Alleles of Class I and II

The epitopes of selected peptides were analyzed for the number of HLA alleles they are predicted to bind using the NetMHCpan 4.1 and NetMHCIIpan 4.0 servers for 2915 HLA class I alleles (886 HLA-A, 1412 HLA-B, and 617 HLA-C) and 681 HLA class II alleles (11 HLA-DP, 9 HLA-DQ, and 661 HLA-DR), respectively. Both P2 and P3 exhibited affinity for many HLA class I alleles. Specifically, P2 was predicted to interact with 839 alleles, while P3 could interact with 840 alleles. P2 and P3 were predicted to bind within the HLA class II alleles with 112 and 164 alleles. On the other hand, P1 showed binding with a total of 373 class II alleles, making it interact with the highest number of HLA among the selected peptides while displaying binding with the lowest count of class I HLA alleles (358) (Table 5).

### 3.4. Interaction Analysis of Peptides with HLA Class I and II Alleles

Due to the closed groove of HLA class I molecules, the nonameric epitopes within the identified peptides were docked with HLA molecules. On the other hand, as class II molecules have an open binding groove, all peptides were docked as they are. The MODPEP server generated three-dimensional structures of the epitopes and identified peptides. Before docking, the peptide structures were refined, and energy minimization was performed.

**Figure 1:**
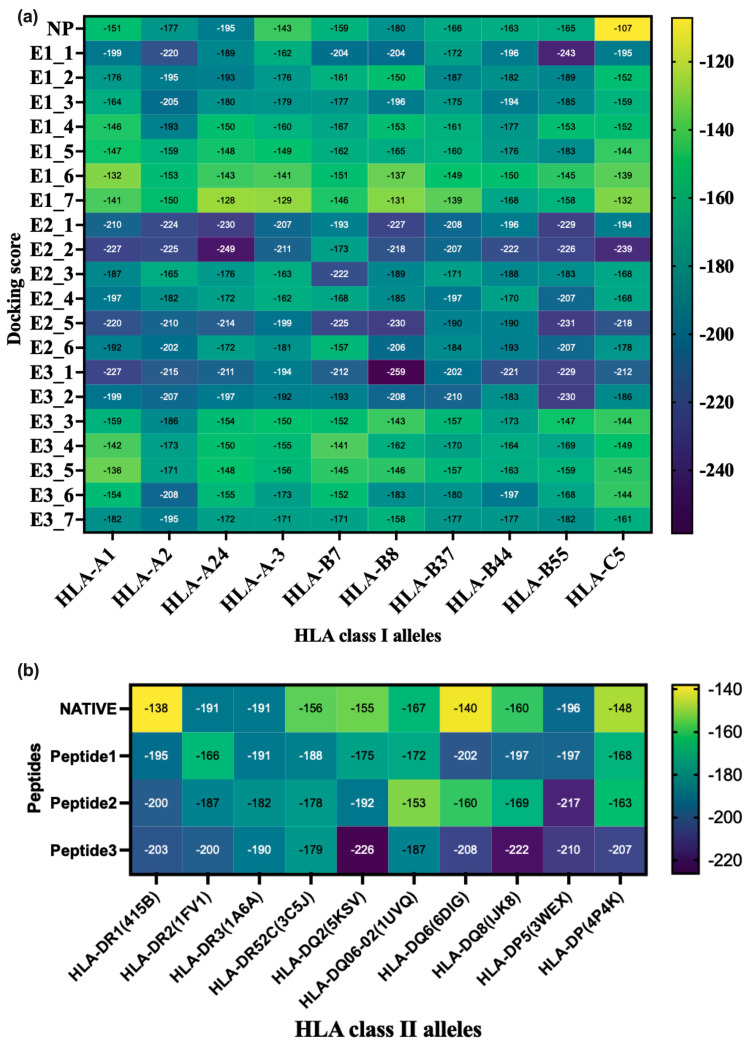
Heat map representing docking score of (**a**) class I HLA alleles and CD8^+^ epitopes and (**b**) class II HLA alleles and peptides.

HPEPDOCK-2.0 was employed for the docking process, where the docking energy score represents the binding affinity of the protein–peptide complex. The docking score of the naturally bound peptide was a reference. Ten HLA class I molecules were docked with both NP and test peptides, and most epitopes demonstrated favorable binding interactions (Figure 1a). All six epitopes associated with peptide 2 (except E2_6: HLA-B7) have shown higher binding energy than the corresponding naturally bound peptide for ten selected HLA class I (Figure 1a). Two epitopes (E3_1, E3_2) of peptide 3 also have higher binding energy for ten HLA class I compared to respective naturally bound peptides (Figure 1a). Three epitopes (E1_1, E1_2, and E1_1, E1_3) of peptide 1 were found to have higher binding energy for nine HLA class I (Figure 1a). Most other epitopes belonging to peptides 1 and 3 have binding energy either higher than or close to naturally bound peptides for different HLA alleles (Figure 1a). For class II, P2 have higher binding energy than the corresponding naturally bound peptides for ten selected HLA class II alleles (Figure 1b). P1 and P3 showed higher binding energy for eight and seven HLA class II alleles (Figure 1b).

### 3.5. Estimated Responses of Peptides across Various Continents

**Figure 2:**
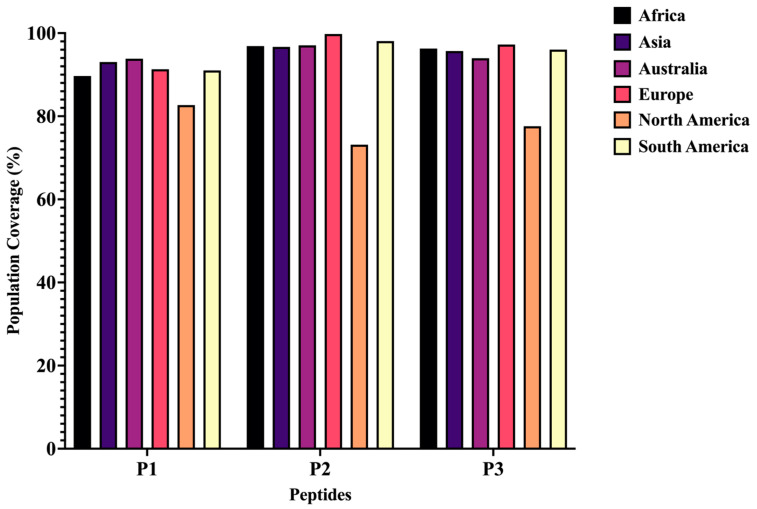
Population coverage of peptides for six continents: Africa, Asia, Australia, Europe, North America, and South America.

It is important to note that adequate population coverage observed for an epitope–HLA complex in one region may not translate to the same coverage in another region. In this study, the population coverage of the three selected N peptides (P1, P2, and P3) was evaluated for each of the six continents: Asia, Europe, North America, Africa, South America, and Australia. Regarding the P1 peptide, four continents (Africa, Asia, Europe, and South America) displayed population coverage higher than 90%, while others had coverage below 90% but above 85%, such as North America (82.69%) and Africa (89.66%). For the P2 peptide, all continents except North America (73.16%) had more than 90% population coverage. Most continents for the P3 peptide demonstrated over 95% population coverage across all continents, except for North America (77.6%) (Figure 2).

### 3.6. Conservation Analysis of Peptides among Different Hantavirus Strains

The analysis was completed to find the sequence identity of the selected PUUV N protein peptides with reference sequences of DOBV, HNTV, Sin Nombre (SNV), and Andes (ANDV). We found a sequence identity across different strains ranging between 83% and 100% (Table 6).

DOBV had an identity ranging from 83% to 92%. In contrast, a P2 peptide of HNTV had 100% with P2. P1 and P3 of PUUV displayed more than 85% in HNTV. Furthermore, the SNV and ANDV had more than 90% identity with the peptide sequences of PUUV.

### 3.7. Structural Analysis of Immunogenic Peptide Construct and Identification of Discontinuous B Cell Epitopes

**Figure 3:**
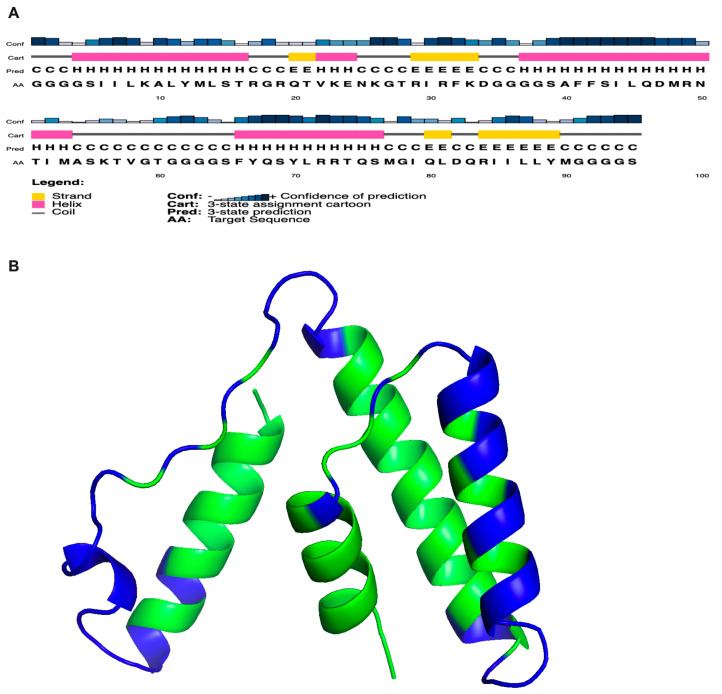
(**A**) The Psipred predicted secondary structure of the immunogenic construct depicting the helical and beta sheet regions. The pink sections represent helices, whereas the yellow regions represent beta strands. (**B**) The shown structure illustrates the three–dimensional arrangement of the immunogenic construct, which includes discontinuous B cell epitopes highlighted in blue.

The generated immunogenic peptide was composed of 95-amino acid residue, which contains three selected peptides and flexible linkers (GGGGS) (Figure 3A). The Robetta-web-server-generated structure with peptides in the order of P1, P2, and P3 showed 95.9% amino acid residues in the favourable region in the Ramachandran plot (Supplementary Figure S1). Based on the results of the Verify3D analysis, it can be concluded that the chosen immunogenic peptide structure is compatible with its amino acid sequence, as 84.21% of the amino acid residues had an average 3D-1D score of ≥0.1, which is higher than the minimum score of 80%. Additionally, after completing the ERRAT study, a comprehensive quality factor of 93.97% was obtained (Supplementary Figure S1). In addition, the immunogenic peptide construct was determined to have a high likelihood of being an antigen (0.6203) and was confirmed to be non-allergenic according to Vaxijen v2.0 and AllerTOP v2.0, respectively. The secondary structure predicted via Psipred showcases a robust confidence interval (Figure 3A), effectively illustrating the domains of alpha helices and beta folds. The discontinuous B cell epitopes predicted using Discotope 2.0 are mapped in the three-dimensional structure of the construct (Figure 3B).

### 3.8. Interaction Analysis between the Immunogenic Peptide Construct and TLR-4

**Figure 4:**
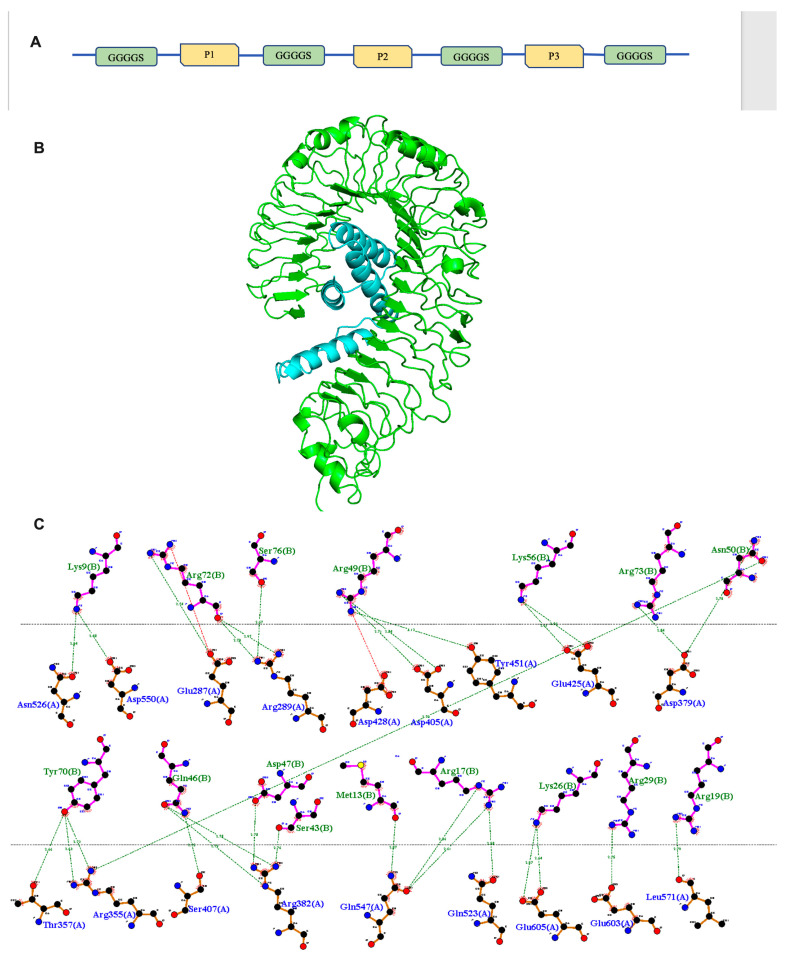
(**A**) The peptide construct is depicted schematically, with linkers connecting the different peptides. (**B**)Three-dimensional representation of the TLR4–peptide docked complex. (**C**) Two-dimensional plot of hydrogen bonding interactions and active residues involved (green and blue depict active residues of peptide and TLR4, respectively).

The immunogenic peptide construct and the naturally bound peptide were subjected to protein–protein docking with TLR-4 using the Cluspro 2.0 server. The naturally bound peptide, when redocked with TLR-4, displayed binding energy −790.7 kcal/mol. The TLR-4 protein was successfully docked with the construct, resulting in a binding energy of −906.8 kcal/mol, which displays a higher binding energy when compared to the naturally bound peptide complex. The docked complex is shown in Figure 4B. Hydrogen bond interactions between various residues of immunogenic peptide construct and TLR-4 are shown in Figure 4C, respectively.

### 3.9. Stability Analysis of TLR-4 and Peptide Construct Based on Molecular Simulation

**Figure 5:**
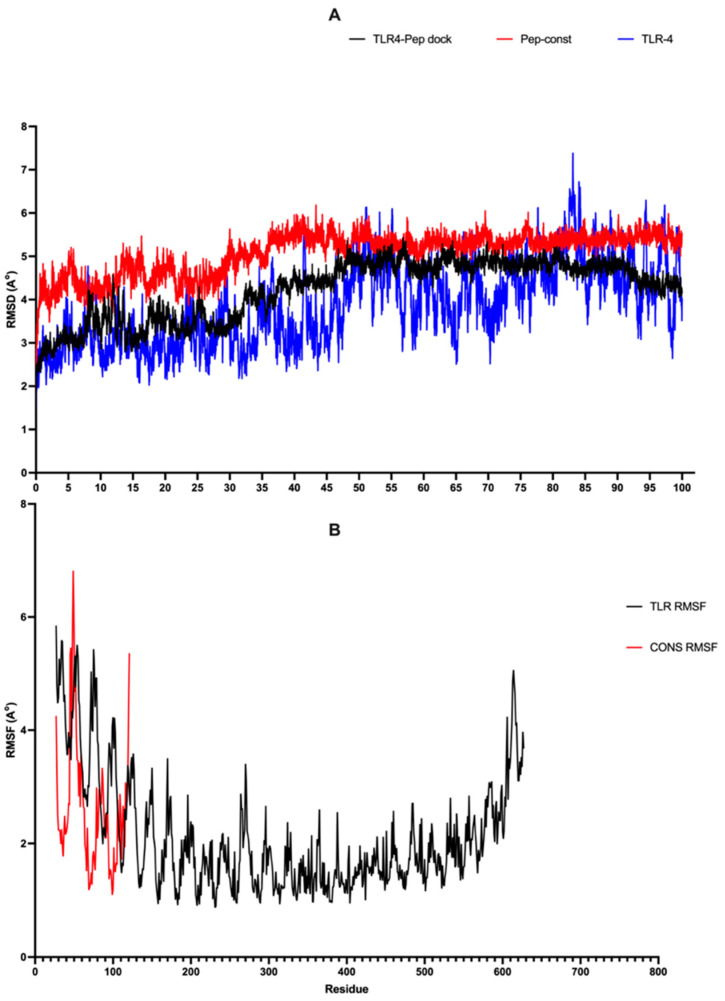
(**A**) RMSD plot of TLR4, Pep-cons (Peptide construct), and TLR4-Pep (TLR4–peptide docked complex). (**B**) RMSF plot of the docked TLR4–peptide complex.

The topology files of TLR-4 and peptide construct and the docked complex were created through the charmm27 force field in the TIP3P water model for molecular simulation analysis. The MD trajectories were used to recover the RMSD and RMSF plots of all molecules. Peptide constructs showed RMSD fluctuation between 28 ns and 48 ns (2–6 Å) when run for around 100 ns, indicating that the peptide construct is unstable for 20 ns. TLR-4 exhibited a deviation from 48 ns to 55 ns and a minor fluctuation of 3–4 ns from 82 ns to 85 ns throughout the simulation. The docked complex in the graph depicted an average RMSD of 4.23 ± 0.69 and showed a deviation from 30 ns to 45 ns for 2–4.5 Å, which concludes that upon the interaction of TLR-4 with peptide construct, the stability of TLR4 increases (Figure 5A). Furthermore, the RMSF plot analyzes the variation in stability of the complex by examining the fluctuations at each amino acid residue. The RMSF graphs displayed a mean RMSF value of 2.10 ± 1.01 and 2.62 ± 1.13 Å for TLR-4 and the immunogenic peptide construct, respectively, present in the complex (Figure 5B). Core residues of TLR-4 and peptide construct were showing fluctuations up to 6.7 Å, and the terminal residues showed mild fluctuations. MD trajectories have provided a description of the stability and compactness of proteins inside of the complex over the course of the simulation.

## 4. Discussion

Orthohantaviruses are zoonotic viruses that naturally persist in some reservoir species through prolonged infection. The mortality of HFRS is low overall but ranges from 0.43% to 15% depending on the infecting virus strain [47], and the mortality associated with HPS is 40–50% [48]. Europe records over 10,000 clinical cases of orthohantavirus disease each year, with case fatality rates varying from less than 1% to over 10% [49]. In 2020, there were 833 documented cases of orthohantavirus infection in the US, with a death rate of 35% [50]. A multi-institutional study [51] has identified 28 cases of infection among chronic renal disease patients, warehouse employees, and members of the Irula tribe who were involved in rat traps in the Vellore region of Tamil Nadu, marking the first report of evidence of orthohantavirus circulation in India. Diagnosis and treatment/prevention are still challenging in controlling the orthohantavirus infection. Ribavarin was shown to be effective only when used in the early stages of infection [7]. Currently, an inactivated orthohantavirus vaccine has been granted licenses only in Korea and China. Ref. [7], while DNA vaccines are still in the early phases of clinical trials. Past exposure to the vaccinia virus was found to hamper the effectiveness of neutralizing antibodies in the volunteers [7].

Immunoinformatic approaches were employed to design an effective immunogenic peptide construct against orthohantavirus [23,52,53]. It was concluded that multiple glycoprotein and N protein peptides contain epitopes with a potential for vaccine design [54]. Joshi and colleagues [52] identified a T cell epitope-based vaccine candidate for orthohantavirus, although their study lacked conservation across multiple strains. However, these studies did not include the prediction of the conserved region that could help deal with different strains worldwide. So, in our studies, after retrieving the data from the NCBI database of one hundred twenty-three protein sequences (sixty-four after removing redundancy), conserved regions were analyzed using the tool AVANA [29], which displayed regions with >90% conservancy. Eliminating the sequences with potential autoimmune responses is crucial as it may trigger autoimmune and cross-reactivity responses, potentially leading to vaccine failure. Vaccination itself has been known to induce autoimmune responses. For instance, Sasaki et al. reported that the influenza vaccine could lead to clinical symptoms of autoimmune diseases like Autoimmune Hepatitis (AIH) [55]. Other studies have also linked vaccines to autoimmune diseases, such as Multiple Sclerosis (MS) and Guillain–Barre syndrome (GBS) [56,57]. The recently developed Moderna COVID-19 vaccination has also been linked to autoimmune illnesses, and it is believed that the vaccine affects self-tolerance and induces autoimmune reactions through cross-reactivity [58]. All of the peptides were screened for autoimmunity using Blastp, and the peptides containing autoimmune regions were not considered for further studies.

Discovering peptides that can attach to HLAs is crucial for activating T cell response. The extensive variation or polymorphism hinders the selection of epitopes that have the potential to interact with diverse HLA alleles in one of the peptide-based studies performed for orthohantavirus [52]. The NetMHCpan 4.1 [30] and NetMHCIIpan 4.0 [59] servers provide an opportunity to define HLA based on a large number of HLA alleles. The three selected peptides were predicted to interact with 358, 839, and 840 HLA class I and 337, 112, and 164 HLA class II molecules. Furthermore, it is impactful to have peptides that contain multiple T and B cell epitopes to cover both humoral and cellular immune responses. Considering this fact, three peptides of PUUV N protein containing multiple CD8^+^, CD4^+^ T cell, and B cell epitopes were selected. By conducting molecular docking and population coverage analyses, we better understood the HLA promiscuity of the selected peptides.

The analysis of population coverage has been performed using the IEDB population coverage tool [20,60]. The present study also analyzed population coverage on six continents by utilizing the IEDB population coverage analysis tool. P3 demonstrated an average population coverage of over 95%. Most peptides have demonstrated population coverage of over 90% in most continents. All three peptides displayed lower coverage for North America. There are similar studies [17,61] where the population coverage for some peptides was shown to be lower in North America than in other continents. The population coverage of peptides could be limited due to the absence of orthohantavirus-binding-dominant HLA alleles in North America in the peptides selected in our study. Molecular docking is used to predict the preferred orientation of a molecule when bound to another in a stable complex. Docking scores of most of the epitopes and peptides were found to be higher than or in the range of scores of naturally bound peptides, indicating the favorable presentation of peptides to HLA class I and II MHC to activate T cell immune response. The result is in agreement with previous studies on peptide–HLA interactions of Dengue [17]. Ismail and colleagues [23] also identified experimentally determined epitopes from the ViPR database and demonstrated their interaction with HLA molecules. We observed the significant sequence identity across the N proteins of different orthohantavirus strains with our final peptides of PUUV. Comparative sequence analysis of the selected peptides with other strain protein sequences revealed substantial identity, ranging between 83 and 100%. The immunogenic peptide construct was generated with the help of linkers and used further for mapping B cell epitopes and TLR docking. Precisely pinpointing the location of B cell epitopes holds crucial significance across various biomedical endeavors, including the strategic design of vaccines, the advancement of disease diagnostics, and the formulation of immunotherapeutics.

TLR-4 detects viral single-stranded RNA and triggers an immune response against the virus [60]. A peptide construct containing multiple SARS-CoV-2 has shown a high docking score with TLR-4 and TLR-3 [61,62]. Furthermore, molecular simulation assessment indicated more stable interaction with TLR-4 than TLR-3. Other studies have shown the interaction of TLR-4 with the peptide construct of SARS-CoV-2 and Dengue virus [63]. In line with these data, we have found that the peptide constructs containing three peptides and linkers had higher docking scores than natural ligands with TLR-4. In similar studies [64,65], the docking score between the construct and TLR4 was notably higher at −1268 kcal/mol and −1035.2 kcal/mol than in our complex (−906.8 kcal/mol). It is important to note that a score surpassing that of the naturally bound peptide does not definitively categorize the construct as a superagonist; rather, it indicates comparable binding energy with the naturally bound peptide. To strengthen our findings, future research should include rigorous in vitro studies for more conclusive evidence. Chichili et al. [66] concluded that the Gly-rich linkers are naturally occurring separators, connecting the domains within the proteins while allowing discrete functions of the domains. In another study [67], Cuspoca et al. stated that the linkers could aid the antigen presentation to HLA molecules. Therefore, we expect that linkers themselves will not typically interrupt the internalization and processing of peptides by the HLA molecules. In an experimental study [68], Kim et al. have demonstrated that the N protein of influenza virus directly binds to TLR2 and TLR4 and induces efficient downstream signaling. Hence, it is expected that the construct generated by joining peptides of N protein of PUUV orthohantavirus harboring immunogenic epitopes may interact with TLR-4 in vivo. The RMSF and RMSD values obtained during the molecular dynamics study showed that the docked complex is stable.

The three selected peptides were not previously reported as such, according to IEDB, which provides information on previous experimentally validated epitopes. Researchers found that peptides ILLKALYML (part of peptide 1) and FYQSYLRRT (part of peptide 3) elicited weak CD8^+^ immune responses in HFRS patients [69]. A peptide, ILQDMRNTI (P2), was considered the only nonameric epitope for N-specific CD8^+^ T lymphocytes in BALB/c mice, and it produced IFN-γ at a rate of 0.14% [70]. The HTNV CD8^+^ T cell epitope (ILQDMRNTI) found in BALB/c mice can also trigger a robust CTL response in HLA-A2.1-infected humans who have successfully recovered from HTNV infection [70]. Further, immunopeptidome profiling of selected peptides in PUUV-infected human microvascular endothelial cells or macrophages will be an interesting analysis to perform in the future.

## 5. Conclusions

Three peptides were obtained using consensus-based prediction methods. These peptides contained numerous CD4^+^ T cell and CD8^+^ T cell epitopes. Prediction algorithms, coverage of the population, and molecular docking data show that the peptides have a high potential to interact with a diverse spectrum of HLA molecules. After joining these three peptides with linkers, the generated peptide construct showed a strong affinity for TLR-4 during docking and simulation assessment. Our data suggest that before selecting the peptide as a possible diagnostic or vaccine candidate against the orthohantavirus, it will be beneficial to complete in silico validation of selected peptides.

## Figures and Tables

**Table 1:** Conserved peptide of *Puumala orthohantavirus nucleocapsid* protein.

Conserved Peptides	Location	Length
MSDLTDIQE	1–9	9
VARQKLKDAE	20–29	10
DPDDVNKNTLQARQQTVSALEDKLAD	35–60	26
MADAVSRKKMDTKPTDPTGIEPD	65–87	23
SLRYGNVLDVNAIDIEEPSGQTADWYTIGVYVIGFT	90–130	36
PIILKALYMLSTRGRQTVKENKGTRIRFKDDTSFED	132–167	36
KHLYVSMPTAQSTMKAEELTPGRFRTIVCGLFPTQIQVRNIMSPVMGVIGFSFFVKDW	175–232	58
KECPFIKPE	242–250	9
VLDKNHVADIDKLIDYAAS	276–294	19
PNAPWVFACAPDRCPPTCIY	306–325	20
AGMAELGAFFSILQDMRNTIMASKTVGTA	327–355	29
SSFYQSYLRRTQSMGIQLDQRIILLYMLEWGKEMVDHFHLGDDMDPELRGLAQ	363–415	53
LIDQKVKEISNQEPLKI	417–433	17

**Table 2:** *Nucleocapsid* peptides containing multiple CD8^+^ T cell epitopes.

Peptides	Location	Length	Epitope Numbers
DVNKNTLQARQQTVSALEDK	38–57	20	6
LRYGNVLDVNAIDIEPSGQTADWYTIGVYV	96–126	30	9
IILKALYMLSTRGRQTVKKENKGTRIRFKDDTSF	133–165	34	8
HLYVSMPTAQSTMKAEELTPGRFRTIVCGLFPTQIQVRNIMSPVMGVIGFSFFVK	176–230	55	19
GMAELGAFFSILQDMRNTIMASKTVGTA	328–355	28	10
SSFYQSYLRRTQSMGIQLDQRIILLYMLEWGKEMVDHFHLGDDMDPELRGL	363–413	51	16
LIDQKVKEISNQEPLKI	417–433	17	5

**Table 3:** *Nucleocapsid* peptides containing multiple CD4^+^ T cell epitopes.

Peptides	Location	Length	Epitope Numbers
NTLQARQQTVSALEDK	42–57	16	2
GNVLDVNAIDIEEPS	93–113	15	1
IILKALYMLSTRGRQTVKENKGTRIRFKD	133–161	29	9
AEELTPGRFRTIVCG	190–204	15	1
AFFSILQDMRNTIMASKTVGT	334–354	21	5
FYQSYLRRTQSMGIQLDQRIILLYM	365–389	25	8

**Table 4:** *Nucleocapsid* peptide containing multiple epitopes of both T and B cells.

Peptides	CD8^+^ Epitopes	CD4^+^ Epitopes	B Cell Epitopes
IILKALYMLSTRGRQTVKENKGTRIRFKD (P1)		IILKALYMLSTRGRQ	LSTRGRQTVKENKGTRIR
	IILKALYML (E1_1)	ILKALYMLSTRGRQT	
	KALYMLSTR (E1_2)	LKALYMLSTRGRQTV	
	LYMLSTRGR (E1_3)	KALYMLSTRGRQTVK	
	LSTRGRQTV (E1_4)	ALYMLSTRGRQTVKE	
	STRGRQTVK (E1_5)	RGRQTVKENKGTRIR	
	GRQTVKENK (E1_6)	GRQTVKENKGTRIRF	
	TVKENKGTR (E1_7)	RQTVKENKGTRIRFK	
		QTVKENKGTRIRFKD	
AFFSILQDMRNTIMASKTVGT (P2)	AFFSILQDM (E2_1)	AFFSILQDMRNTIMA	AFFSILQDMRNTIMASKT
	FFSILQDMR (E2_2)	FFSILQDMRNTIMAS	
	ILQDMRNTI (E2_3)	FSILQDMRNTIMASK	
	LQDMRNTIM (E2_4)	LQDMRNTIMASKTVG	
	MRNTIMASK (E2_5)	QDMRNTIMASKTVGT	
	NTIMASKTV (E2_6)		
FYQSYLRRTQSMGIQLDQRIILLYM (P3)		FYQSYLRRTQSMGIQ	YQSYLRRTQSMGIQLDQR
	SYLRRTQSM (E3_1)	YQSYLRRTQSMGIQL	
	RTQSMGIQL (E3_2)	QSYLRRTQSMGIQLD	
	MGIQLDQRI (E3_3)	SYLRRTQSMGIQLDQ	
	IQLDQRIIL (E3_4)	RTQSMGIQLDQRIIL	
	QLDQRIILL (E3_5)	TQSMGIQLDQRIILL	
	LDQRIILLY (E3_6)	QSMGIQLDQRIILLY	
	DQRIILLYM (E3_7)	SMGIQLDQRIILLYM	

**Table 5:** The number of unique human leukocyte antigen (HLA) class I and II predicted to bind with the selected *Nucleocapsid* peptides.

Peptides	HLA Class I (CD8^+^ T Cell Epitopes)			HLA Class II (CD4^+^ T Cell Epitopes)		
	HLA-A	HLA-B	HLA-C	HLA-DP	HLA-DQ	HLA-DR
	886	1412	617	11	9	661
IILKALYMLSTRGRQTVKENKGTRIRFKD (P1)	264	46	48	0	1	372
AFFSILQDMRNTIMASKTVGT (P2)	532	304	3	5	0	107
FYQSYLRRTQSMGIQLDQRIILLYM (P3)	560	279	1	0	0	164

The red colour is the number of alleles that were considered in prediction tools to predict the T cell epitopes.

**Table 6:** The identity analysis of final peptide sequences (P1, P2, and P3) of PUUV and other orthohantavirus strains.

Hantavirus	IILKALYMLSTRGRQTVKENKGTRIRFKD (P1)	AFFSILQDMRNTIMASKTVGT (P2)	FYQSYLRRTQSMGIQLDQRIILLYM (P3)
HNTV	ILLKALYMLTTRGRQTTKDNKGTRIRFKD (86%)	AFFSILQDMRNTIMASKTVGT (100%)	FYQSYLRRTQSMGIQLGQRIIVLFM (88%)
DOBV	ILLKALYMLTTRGRQTTKDNKGMRIRFKD (83%)	AFFAVLQDMRNTIMASKTIGT (86%)	FYQSYLRRTQSMGIQLDQRIIVLFM (92%)
SNV	IILKALYMLSTRGRQTIKENKGTRIRFKD (97%)	AFFAILQDMRNTIMASKSVGT (90%)	FYQSYLRRTQSMGIQLDQKIIILYM (92%)
ANDV	IILKALYMLSTRGRQTVKDNKGTRIRFKD (97%)	AFFSILQDMRNTIMASKSVGT (95%)	FYQSYLRRTQSMGIQLDQKIIILYM (92%)

Highlighted in red within the table are amino acids distinct from the PUUV peptides.

## Data Availability

The original contributions presented in the study are included in the article/supplementary material, further inquiries can be directed to the corresponding author/s.

## Supplementary Materials

The following supporting information can be downloaded at: https://www.mdpi.com/article/10.3390/v16071030/s1, Table S1: Conserved peptide fragments *Puumala orthohantavirus* nucleocapsid protein having peptides resembling to human protein; Table S2: Peptides containing CD8^+^ T cell epitopes; Table S3: Peptides containing CD4^+^ T cell epitopes; Figure S1: (A): Verify3D result of the model. The plot displays 84.21% residues with averaged 3D-1D score > 0.1. (B): Ramachandran plot of the construct model is determined by Procheck. (C): The ERRAT analysis showing the quality factor of 93.97% of the refined 3D immunogenic peptide construct.
