# Supplementary material for: Designing a Conserved Immunogenic Peptide Construct from the Nucleocapsid Protein of Puumala orthohantavirus
Source: Viruses. 2024 Jun 26;16(7):1030. doi: 10.3390/v16071030 (PMC11281540; doi:10.3390/v16071030)
Supplement: Supplementary file 1 [file viruses-16-01030-s001.zip › viruses-2988064-supplementary.pdf]

Table 1. Conserved peptide fragments Puumala orthohantavirus nucleocapsid protein having peptides resembling to human protein.

Peptide	Location	Length	Human protein
MSDLTDIQE	1-9	9	None
ITRHEQQLVVARQKLKDAE	11-29	19	Immunoglobulin Heavy chain
DPDDVNKNTLQARQQTVSALEDKLAD	35-60	26	None
MADAVSRKKMDTKPTDPTGIEPDDHLKERS-SLRYGNVLDVNAIDI-EEPSGQTADWYTIGVYVIGFT	65-130	66	Zinc finger 33A
PIILKALYMLSTRGRQTVKENKGTRI-RFKDDTSFEDING-IRRPKHLYVSMPTAQSTMKAEELTPGRFRTIVCG LFPTQIQVRNIMSPVMGVIGFSFFVKDW	132-232	101	APE2 protein, DNA-(apurinic or apyrimidinic site) endonuclease 2 isoform 1, DNA-apurinic or apyrimidinic site lyase 2
KECPFIKPE	242-250	9	None
VLDKNHVADIDKLIDYAASGDPTSPD	276-301	26	Immunoglobulin Heavy chain
PNAPWVFACAPDRCPPTCIY	306-325	20	None
AGMAELGAFFSILQDMRNTMASKTVGTAEEK-LKKKSSFYQSYLRRTQSMGIQLDQRIILLYM-LEWGKEMVDHFHLGDDMDPELRGLAQ	327-415	88	Cardiac alpha-myosin heavy chain
LIDQKVKEISNQEPLKI	417-433	17	None

*The regions in red color indicate the part of the peptides that resemble to mentioned human protein.

Table 2. Peptides containing CD8+ T cell epitopes.

Peptides	Epitopes
VARQKLKDAE	VARQKLKDA
PDDVNKNTLQARQQTVSALEDKLAD	DVNKNTLQA, VNKNTLQAR, TLQARQQTV, QARQQTVSA, ARQQTVSAL, QTVSALEDK
SLRYGNVLDVNAIDIEEPSGQTADWYTIGVYVIGFT	LRYGNVLDV, VLDVNAIDI, IEEPSGQTA, VLDV-NAIDI, EPSGQTADW, GQTADWYTI, TADWYTIGV, ADWYTIGVY, DWYTIGVYV
PIILKALYMLSTRGRQTVKENKGTRIRFKDDTSFED	IILKALYML, KALYMLSTR, LYMLSTRGR, LSTRGRQTV, STRGRQTVK, GRQTVKENK, TVKENKGTR, IRFKDDTSF
KHLYVSMPTAQSTMKAEELTPGRFRTIVCGLF-PTQIQVRNIMSPVMGVIGFSFFVKDW	HLYVSMPTA, SMPTAQSTM, AEELTPGRF, LTPGRFRTI, TPGRFRTIV, RFRTIVCGL, FRTIVCGLF, GLFPTQIQV, LFPTQIQVR, TQIQVRNIM, IQVRNIMSP, QVRNIMSPV, VRNIMSPVM, NIMSPVMGV, IMSPVMGVI, SPVMGVIGF, VMGVIGFSF, GVIGFSFFV, VIGFSFFVK
AGMAELGAFFSILQDMRNTIMASKTVGTA	GMAELGAFF, AELGAFFSI, AFFSILQDM, FFSILQDMR, ILQDMRNTI, LQDMRNTIM, QDMRNTIMA, MRNTIMASK, NTIMASKTV, MASKTVGTA
SSFYQSYLRRTQSMGIQLDQRIILLYM-LEWGKEMVDHFHLGDDMDPELRGLAQ	SSFYQSYLR, SFYQSYLRR, SYLRRTQSM, RTQSMGIQL, MGIQLDQRI, IQLDQRIIL, QLDQRIILL, LDQRIILLY, DQRIILLYM, QRIILLYML, IILLYMLEW, YMLEWGKEM,

	KEMVDHFHL, DHFHLGDDM, LGDDMDPEL, DMDPELRGL
LIDQKVKEISNQEPLKI	LIDQKVKEI, KVKEISNQE, KEISNQEPL, EISNQEPLK, ISNQEPLKI

Table 3. Peptides containing CD4⁺ T cell epitopes.

Peptides	Epitopes
DVNKNTLQARQQTVSALEDK	NTLQARQQTVSALED, TLQARQQTVSALEDK
LRYGNVLDVNAIDIEPSGQTADWYTIGVYV	GNVLDVNAIDIEEPS
IILKALYMLSTRGRQTVKKENKGTRIRFKDDTSF	IILKALYMLSTRGRQ, ILKALYMLSTRGRQT, LKALYMLSTRGRQTV, KALYMLSTRGRQTVK, ALYMLSTRGRQTVKE, RGRQTVKENKGTRIR, GRQTVKENKGTRIRF, RQTVKENKGTRIRFK, QTVKENKGTRIRFKD
HLYVSMPTAQSTMKAEELTPGRFRTIVCGLFPTQIQVRNIMSPVMGVIGFSFFVK	AEELTPGRFRTIVCG
GMAELGAFFSILQDMRNTIMASKTVGTA	AFFSILQDMRNTIMA, FFSILQDMRNTIMAS, FSILQDMRNTIMASK, LQDMRNTIMASKTVG, QDMRNTIMASKTVGT
SSFYQSYLRRTQSMGIQLDQRIILLYMLEWGKEMVDHFHLGDDMDPELRGL	FYQSYLRRTQSMGIQ, YQSYLRRTQSMGIQL, QSYLRRTQSMGIQLD, SYLRRTQSMGIQLDQ, RTQSMGIQLDQRIIL, TQSMGIQLDQRIILL, QSMGIQLDQRIILLY, SMGIQLDQRIILLYM

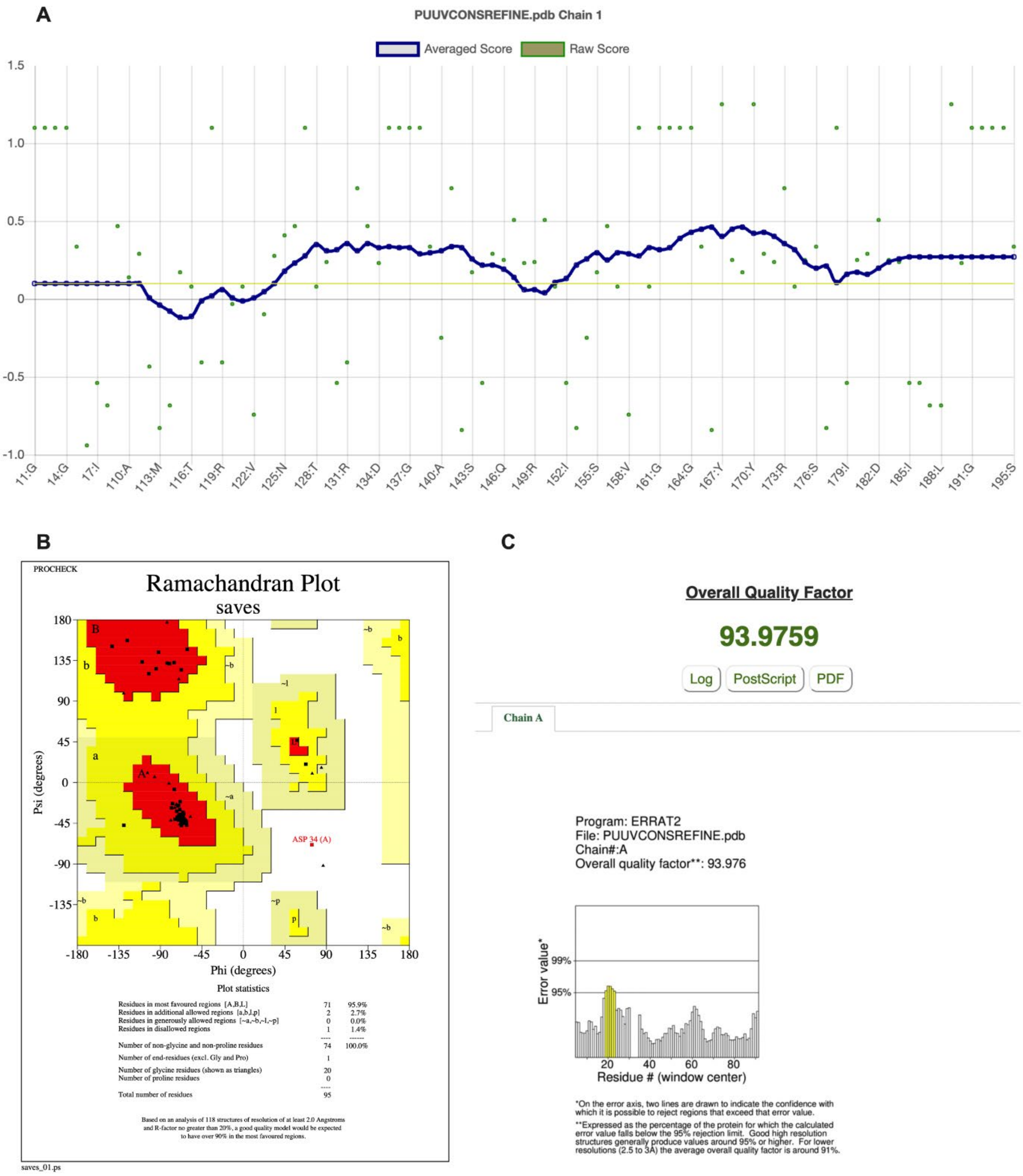

Figure S1. (A): Verify3D result of the model. The plot displays 84.21% residues with averaged 3D-1D score >0.1. **(B):** Ramachandran plot of the construct model is determined by Procheck. **(C):** The ERRAT analysis showing the quality factor of 93.97% of the refined 3D immunogenic peptide construct.
